# *catena*-Poly[[[bis­(*N*,*N*-dimethyl­formamide)iron(II)]-{μ-2,2′-bis­(diphenyl­phosphino­yl)-*N*,*N*′-[(1*R*,2*R*)-cyclo­hexane-1,2-di­yl]dibenzamide}] bis­(perchlorate) *N*,*N*-dimethyl­formamide disolvate]

**DOI:** 10.1107/S1600536809045188

**Published:** 2009-11-04

**Authors:** Grant R. Ferrell, Curtis Moore, Arnold L. Rheingold, Christopher J. A. Daley

**Affiliations:** a Department of Chemistry and Biochemistry, University of San Diego, 5998 Alcalá Park, San Diego, CA 92110, USA; b Department of Chemistry and Biochemistry, University of California, San Diego, 9500 Gilman Drive, La Jolla, CA 92093, USA

## Abstract

The title extended solid coordination compound, {[Fe(C_44_H_40_N_2_O_4_P_2_)(C_3_H_7_NO)_2_](ClO_4_)_2_·2C_3_H_7_NO}_*n*_, was crystallized un­ex­pectedly from the reaction mixture containing the Trost ligand (1*R*,2*R*)-(+)-1,2-diamino­cyclo­hexane-*N*,*N*′-bis­(2′-di­phenyl­phosphinobenzo­yl) and Fe(ClO_4_)_2_·6H_2_O in a 1:1 ratio in dimethyl­formamide (DMF) under reflux conditions. The polymeric complex is characterized by Fe^II^ metal centers that are coordinated by two oxidized Trost ligands, each coordinated in a bidentate fashion in a square plane, along with two DMF mol­ecules above and below the plane [average Fe—O_DMF_ = 2.086 (4) Å], forming an overall pseudo-octa­hedral geometry. The Trost ligand binds adjacent Fe^II^ centers, each Fe^II^ being bound through the O atom of one of the phosphine oxides [average Fe—O_PPh2_ = 2.115 (4) Å] and the carbonyl O atom of the adjacent amide [average Fe—O_amide_ = 2.192 (3) Å]. Disorder is observed in the co-solvated solvent: there are two DMF mol­ecules per Fe^II^ centre, which were modeled as one DMF mol­ecule with complete occupancy and the other being modeled in two positions with equal occupancy. Disorder was also observed with one of the perchlorate anions, which was modeled in two positions with 0.75:0.25 occupancy.

## Related literature

For a general background to diamidato-bis­(phosphine) ligand systems, see: Trost *et al.* (1994 ▶); Chahan *et al.* (2006 ▶); Burger *et al.* (2003 ▶); Campos *et al.* (2005 ▶). For related structures of iron complexes with bis­(imino­phospho­rane)-bis­(phosphine oxide) ligands, see: Buchard *et al.* (2009 ▶). For Fe-O_Amide_ bond distances, see: Mandal & Que (1997 ▶); Constant *et al.* (1971 ▶); Müller *et al.* (1989 ▶). For Fe—OPPh_2_ bond lengths, see: Buchard *et al.* (2009 ▶); Escriche *et al.* (2006 ▶); For the preparation, see: Gao *et al.* (1996 ▶). 
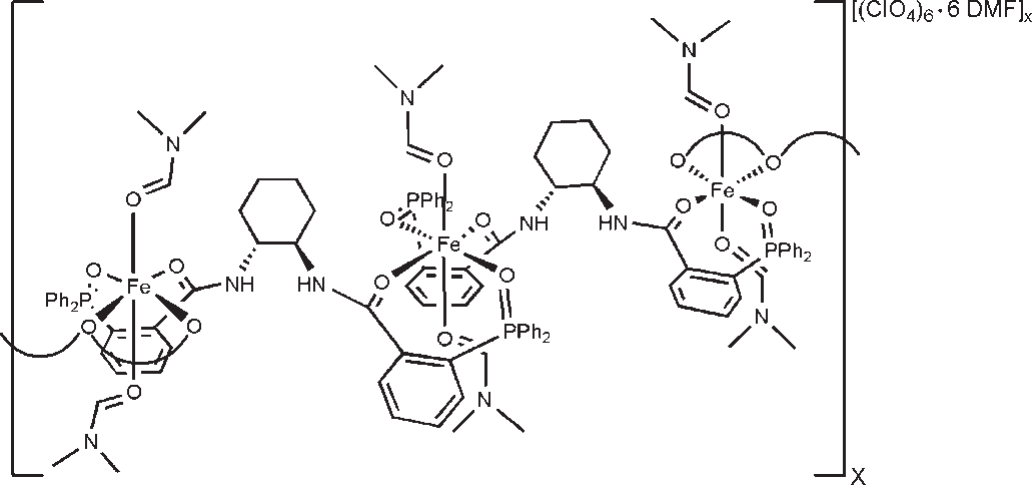


## Experimental

### 

#### Crystal data

[Fe(C_44_H_40_N_2_O_4_P_2_)(C_3_H_7_NO)_2_]·(ClO_4_)_2_·2C_3_H_7_NO
*M* *_r_* = 1269.85
Orthorhombic, 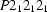

*a* = 10.9725 (4) Å
*b* = 15.4417 (6) Å
*c* = 36.2173 (14) Å
*V* = 6136.4 (4) Å^3^
*Z* = 4
Mo *K*α radiation
μ = 0.46 mm^−1^
*T* = 150 K
0.25 × 0.25 × 0.20 mm

#### Data collection

Bruker APEXII CCD diffractometer
Absorption correction: multi-scan (*SADABS*; Bruker, 2001 ▶) *T*_min_ = 0.895, *T*_max_ = 0.914
41558 measured reflections
12467 independent reflections
9643 reflections with *I* > 2σ(*I*)
*R*_int_ = 0.053

#### Refinement

*R*[*F* ^2^ > 2σ(*F* ^2^)] = 0.069
*wR*(*F* ^2^) = 0.197
*S* = 1.04
12467 reflections
820 parameters
110 restraints
H-atom parameters constrained
Δρ_max_ = 0.90 e Å^−3^
Δρ_min_ = −0.68 e Å^−3^
Absolute structure: Flack (1983 ▶), 5499 Friedel pairs
Flack parameter: 0.01 (2)

### 

Data collection: *APEX2* (Bruker, 2007 ▶); cell refinement: *SAINT* (Bruker, 2007 ▶); data reduction: *SAINT*; program(s) used to solve structure: *SHELXS97* (Sheldrick, 2008 ▶); program(s) used to refine structure: *SHELXL97* (Sheldrick, 2008 ▶); molecular graphics: *ORTEP-3* (Farrugia, 1997 ▶); software used to prepare material for publication: *publCIF* (Westrip, 2009 ▶).

## Supplementary Material

Crystal structure: contains datablocks I, global. DOI: 10.1107/S1600536809045188/br2120sup1.cif

Structure factors: contains datablocks I. DOI: 10.1107/S1600536809045188/br2120Isup2.hkl

Additional supplementary materials:  crystallographic information; 3D view; checkCIF report

## Figures and Tables

**Fig. 1.**
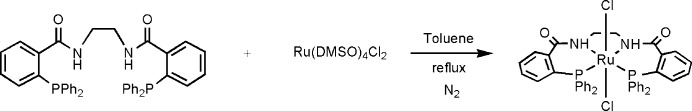
Reported synthesis of a neutral RuII complex containing an achiral aliphatic Trost ligand analogue.

**Fig. 2.**
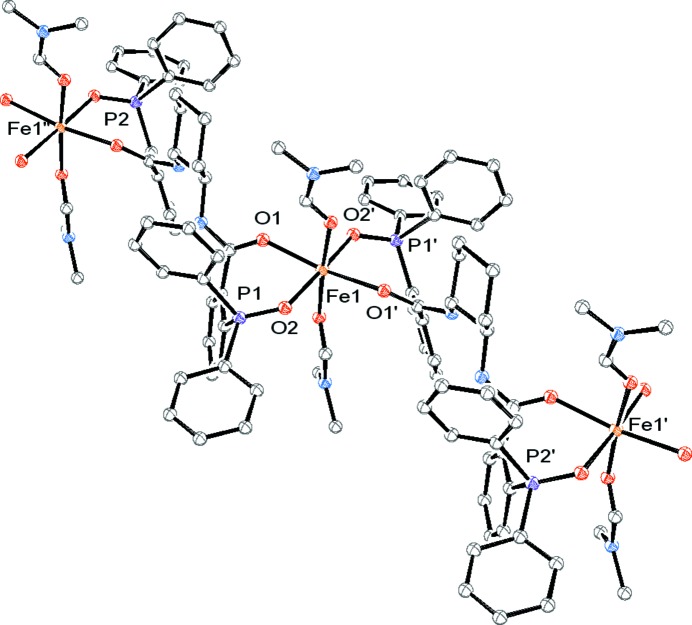
Perspective view of the polymeric cationic portion of the title structure showing three FeII centers and the coordination geometry of the oxidized Trost ligand ((1R,2R)-(+)-1,2- diaminocyclohexane-N,N'-bis(2'-diphenyloxophosphinobenzoyl)) that bridges adjacent FeII centers in the polymer. H-atoms, co-solvated DMF, and ClO4- anions are not displayed for clarity. Thermal ellipsoids are drawn at 30% probability.

**Fig. 3.**
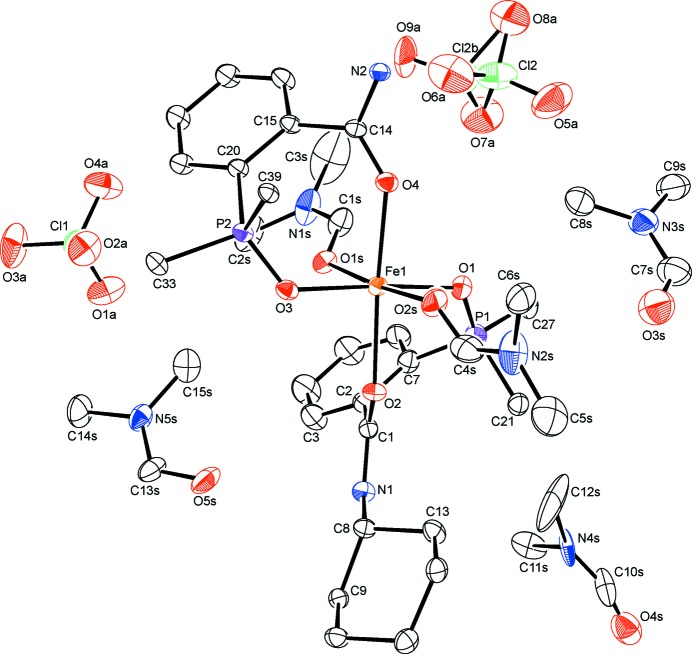
A secondary perspective view showing the asymmetric unit of the title compound including the ClO4- anions and co-solvated DMF molecules. H-atoms and the phenyl ring substituents of the phosphines have been omitted for clarity. Thermal ellipsoids are drawn at 30% probability.

## supplementary crystallographic information

### Comment

In the course of examining metal-amidato systems, we reacted the Trost ligand (1*R*,2*R*)-(+)-1,2- diaminocyclohexane-*N*,*N*'-bis(2'-diphenylphosphinobenzoyl) with Fe(ClO_4_)_2_.6H_2_O in order to form the tetracoordinated Fe^II^ complex using similar conditions reported by Wong (Gao *et al.*, 1996) for the analogous Ru^II^ complex (Figure 1). Using Fe(ClO_4_)_2_.6H_2_O as the metal salt and DMF as the solvent, a pale brown solid was obtained which was recrystallized for *x*-ray analysis.

The structure of the extended cation of [[Fe(µ-(κ^4^-O^1^:*O*^2^, O^1'^: O^2'^ H_2_N_2_(P O)_2_)_2_(DMF)_2_](ClO_4_)_2_.2DMF]*_x_* is shown in Figure 2. It is noted that adventitious water and/or oxygen oxidized the phosphine moieties. Only one isomer of the product was observed; where two oxidized Trost ligands (phosphine oxides) adopt a *trans*-coordination geometry with respect to each other, and two DMF molecules binding to the apical sites forming an octahedral Fe^II^ complex (Figure 3). The ligands bond to Fe^II^ through the amide carbonyl O atom and the O atom of the oxidized PPh_2_ unit. The Fe—OPPh_2_ bond lengths are 2.089 (3) and 2.140 (4) Å, which are in the range of those reported (2.097 (2) - 2.132 (2) Å) for other Fe^II^ phosphine oxide complexes (*e.g.* Buchard *et al.*, 2009 and Escriche *et al.*, 2006).

The Fe-O_Amide_ bond distances (2.187 (3) and 2.197 (3) Å) are longer than those observed in [Fe^II^(BPGm)(O_2_CCH_3_)(CH_3_OH)]BPh_4_ (2.170 (5) Å; BPGm = bis(2-pyridylmethyl)glycinamide: Mandal *et al.*, 1997) and significantly longer than those in Fe—O_DMF_ complexes (2.12 Å average; Constant *et al.*, 1971; Müller *et al.*, 1989), including those in the title structure (Fe—O_DMF_: 2.084 (4) and 2.087 (4) Å). Disorder was observed solely from the co-solvated DMF molecules and one of the perchlorate anions (see Figure 3). The co-solvated DMF was modeled with one molecule at 100% occupancy and the other modeled at two positions (DMF's containing N3s and N4s) with 50:50 occupancy. Bond distances of one DMF molecule were restrained owing to unmodelable disorder. The disordered perchlorate ion (containing Cl2) was modeled in two positions with 75:25 occupancy and some of the thermal parameters were restrained to be similar to each other because of NPD's.

### Experimental

To a 100 ml sidearm flask containing a stirbar and ligand (1*R*,2*R*)-(+)-1,2- diaminocyclohexane-*N*,*N*'-bis(2'-diphenylphosphinobenzoyl) (0.1235 g, 1.787x10^-4^ mol) was added Fe(ClO_4_)_2_.6 H_2_O (0.06650 g, 1.833x10^-4^ mol). The sidearm had a septum placed on it and was evacuated under high vacuum and re-filled with N_2_ (x3) and left under an N_2_ atmosphere. De-oxygenated acetonitrile (dried *via* Innovative Technologies solvent purification system: SPS) was cannulated (25 ml) into the sidearm. The mixture was left unstirred for 4 h and then it was stirred and all solid dissolved leaving a light yellow solution. The septum on the sidearm was replaced with a reflux condenser and gas-inlet adaptor (oven dried overnight), all flushed with N_2_. The reaction mixture was heated to reflux in an oil bath (80–85 °C) and left to stir for 14 h. The solution was cooled to room temperature and the solution was filtered into another sidearm flask. The filtrate solvent was removed under reduced pressure leaving a resulting brown solid (mass) that was transferred to the glovebox for storage. The solid (20 mg) was dissolved in minimal DMF (dried *via* SPS), filtered to remove any undissolved particles, and placed in a 1 dram vial. The vial was placed in a 3 dram vial containing diethyl ether (dried *via* SPS) and the 3 dram vial was sealed to allow vapor diffusion crystallization to occur. After 2 days, oval crystals of the title complex precipitated and one was characterized by *x*-ray crystallography.

### Refinement

Owing to unmodelable disorder, the bond distances on one of the co-solvated molecules of DMF was restrained. Furthermore, one of the co-solvated DMF molecules was disordered 75:25. One of the perchlorates was disordered 50:50 and some of the thermal parameters were restrained to be similar to each other because of NPD's.

### Crystal data

**Table:**

[Fe(C_44_H_40_N_2_O_4_P_2_)(C_3_H_7_NO)_2_]·(ClO_4_)_2_·2C_3_H_7_NO	*F*(000) = 2656
*M**_r_* = 1269.85	*D*_x_ = 1.375 Mg m^−^^3^
Orthorhombic, *P*2_1_2_1_2_1_	Mo *K*α radiation, λ = 0.71073 Å
Hall symbol: P 2ac 2ab	Cell parameters from 9959 reflections
*a* = 10.9725 (4) Å	θ = 2.2–25.6°
*b* = 15.4417 (6) Å	µ = 0.46 mm^−^^1^
*c* = 36.2173 (14) Å	*T* = 150 K
*V* = 6136.4 (4) Å^3^	Block, yellow
*Z* = 4	0.25 × 0.25 × 0.20 mm

### Data collection

**Table:**

Bruker APEXII CCD diffractometer	12467 independent reflections
Radiation source: fine-focus sealed tube	9643 reflections with *I* > 2σ(*I*)
graphite	*R*_int_ = 0.053
φ and ω scans	θ_max_ = 26.4°, θ_min_ = 1.4°
Absorption correction: multi-scan (*SADABS*; Bruker, 2001)	*h* = −13→13
*T*_min_ = 0.895, *T*_max_ = 0.914	*k* = −19→18
41558 measured reflections	*l* = −44→45

### Refinement

**Table:**

Refinement on *F*^2^	Hydrogen site location: inferred from neighbouring sites
Least-squares matrix: full	H-atom parameters constrained
*R*[*F*^2^ > 2σ(*F*^2^)] = 0.069	*w* = 1/[σ^2^(*F*_o_^2^) + (0.1202*P*)^2^ + 2.5012*P*] where *P* = (*F*_o_^2^ + 2*F*_c_^2^)/3
*wR*(*F*^2^) = 0.197	(Δ/σ)_max_ = 0.001
*S* = 1.04	Δρ_max_ = 0.90 e Å^−^^3^
12467 reflections	Δρ_min_ = −0.68 e Å^−^^3^
820 parameters	Extinction correction: *SHELXL97* (Sheldrick, 2008), Fc^*^=kFc[1+0.001xFc^2^λ^3^/sin(2θ)]^-1/4^
110 restraints	Extinction coefficient: 0.0008 (3)
Primary atom site location: structure-invariant direct methods	Absolute structure: Flack (1983), 5499 Friedel pairs
Secondary atom site location: difference Fourier map	Flack parameter: 0.01 (2)

### Special details

**Table:**

Geometry. All s.u.'s (except the s.u. in the dihedral angle between two l.s. planes) are estimated using the full covariance matrix. The cell s.u.'s are taken into account individually in the estimation of s.u.'s in distances, angles and torsion angles; correlations between s.u.'s in cell parameters are only used when they are defined by crystal symmetry. An approximate (isotropic) treatment of cell s.u.'s is used for estimating s.u.'s involving l.s. planes.
Refinement. Refinement of *F*^2^ against ALL reflections. The weighted *R*-factor *wR* and goodness of fit *S* are based on *F*^2^, conventional *R*-factors *R* are based on *F*, with *F* set to zero for negative *F*^2^. The threshold expression of *F*^2^ > 2σ(*F*^2^) is used only for calculating *R*-factors(gt) *etc*. and is not relevant to the choice of reflections for refinement. *R*-factors based on *F*^2^ are statistically about twice as large as those based on *F*, and *R*- factors based on ALL data will be even larger.

### Fractional atomic coordinates and isotropic or equivalent isotropic displacement parameters (Å^2^)

**Table:**

	*x*	*y*	*z*	*U*_iso_*/*U*_eq_	Occ. (<1)
C1	0.3022 (4)	0.4030 (3)	0.86152 (14)	0.0348 (12)	
C2	0.3236 (5)	0.4777 (4)	0.83557 (16)	0.0398 (12)	
C3	0.2628 (6)	0.5532 (4)	0.84174 (19)	0.0558 (17)	
H3	0.2019	0.5557	0.8603	0.067*	
C4	0.2895 (7)	0.6270 (4)	0.8209 (2)	0.071 (2)	
H4	0.2496	0.6803	0.8260	0.085*	
C5	0.3751 (7)	0.6214 (5)	0.7927 (2)	0.067 (2)	
H5	0.3927	0.6709	0.7780	0.080*	
C6	0.4335 (6)	0.5457 (4)	0.78609 (18)	0.0535 (16)	
H6	0.4913	0.5426	0.7666	0.064*	
C7	0.4099 (5)	0.4708 (4)	0.80771 (14)	0.0398 (12)	
C8	0.1502 (4)	0.3087 (3)	0.89115 (13)	0.0311 (11)	
H8	0.1944	0.3167	0.9151	0.037*	
C9	0.0127 (4)	0.3125 (3)	0.89886 (13)	0.0292 (10)	
H9	−0.0317	0.3013	0.8752	0.035*	
C10	−0.0212 (5)	0.2422 (3)	0.92629 (15)	0.0359 (12)	
H10A	0.0203	0.2536	0.9501	0.043*	
H10B	−0.1102	0.2439	0.9307	0.043*	
C11	0.0139 (5)	0.1532 (3)	0.91265 (16)	0.0383 (12)	
H11A	−0.0357	0.1387	0.8907	0.046*	
H11B	−0.0044	0.1100	0.9321	0.046*	
C12	0.1476 (5)	0.1480 (4)	0.90273 (16)	0.0384 (12)	
H12A	0.1651	0.0911	0.8913	0.046*	
H12B	0.1974	0.1531	0.9254	0.046*	
C13	0.1821 (5)	0.2198 (3)	0.87601 (15)	0.0372 (12)	
H13A	0.1388	0.2107	0.8523	0.045*	
H13B	0.2707	0.2171	0.8710	0.045*	
C14	0.8661 (4)	0.4277 (3)	0.90926 (13)	0.0271 (10)	
C15	0.8451 (4)	0.5177 (3)	0.92230 (13)	0.0275 (10)	
C16	0.9171 (5)	0.5839 (3)	0.90780 (15)	0.0385 (12)	
H16	0.9811	0.5697	0.8912	0.046*	
C17	0.8976 (5)	0.6692 (4)	0.91698 (17)	0.0425 (13)	
H17	0.9486	0.7132	0.9071	0.051*	
C18	0.8029 (5)	0.6908 (3)	0.94073 (17)	0.0420 (13)	
H18	0.7885	0.7497	0.9469	0.050*	
C19	0.7292 (4)	0.6265 (3)	0.95554 (15)	0.0348 (11)	
H19	0.6645	0.6418	0.9717	0.042*	
C20	0.7491 (4)	0.5390 (3)	0.94691 (13)	0.0272 (10)	
C21	0.3980 (5)	0.2834 (4)	0.79526 (14)	0.0370 (12)	
C22	0.2800 (6)	0.2909 (5)	0.78274 (17)	0.0523 (16)	
H22	0.2456	0.3466	0.7788	0.063*	
C23	0.2120 (7)	0.2179 (6)	0.77596 (19)	0.067 (2)	
H23	0.1305	0.2235	0.7675	0.080*	
C24	0.2604 (7)	0.1373 (5)	0.78130 (18)	0.063 (2)	
H24	0.2135	0.0870	0.7761	0.076*	
C25	0.3760 (7)	0.1300 (5)	0.79404 (17)	0.0575 (17)	
H25	0.4091	0.0740	0.7981	0.069*	
C26	0.4467 (6)	0.2018 (4)	0.80121 (17)	0.0484 (15)	
H26	0.5277	0.1954	0.8101	0.058*	
C27	0.5632 (6)	0.3843 (4)	0.75390 (17)	0.0515 (15)	
C28	0.4870 (8)	0.3819 (5)	0.72211 (17)	0.0629 (18)	
H28	0.4009	0.3799	0.7247	0.075*	
C29	0.5387 (10)	0.3824 (5)	0.6877 (2)	0.078 (2)	
H29	0.4870	0.3819	0.6667	0.094*	
C30	0.6579 (10)	0.3836 (6)	0.6828 (2)	0.082 (2)	
H30	0.6903	0.3834	0.6585	0.098*	
C31	0.7384 (9)	0.3851 (8)	0.7137 (2)	0.100 (3)	
H31	0.8242	0.3846	0.7103	0.120*	
C32	0.6868 (7)	0.3874 (6)	0.74988 (19)	0.076 (2)	
H32	0.7380	0.3909	0.7710	0.091*	
C33	0.5714 (4)	0.5073 (3)	1.00510 (13)	0.0284 (10)	
C34	0.4438 (5)	0.5076 (3)	1.00321 (15)	0.0362 (12)	
H34	0.4013	0.4818	0.9832	0.043*	
C35	0.3810 (5)	0.5486 (4)	1.03304 (17)	0.0453 (14)	
H35	0.2945	0.5516	1.0328	0.054*	
C36	0.4436 (6)	0.5833 (4)	1.06169 (17)	0.0468 (15)	
H36	0.4003	0.6091	1.0816	0.056*	
C37	0.5691 (6)	0.5815 (3)	1.06238 (16)	0.0445 (14)	
H37	0.6114	0.6061	1.0827	0.053*	
C38	0.6335 (5)	0.5446 (3)	1.03398 (15)	0.0367 (11)	
H38	0.7200	0.5448	1.0343	0.044*	
C39	0.7466 (4)	0.3751 (3)	0.98693 (12)	0.0250 (9)	
C40	0.7016 (4)	0.2908 (3)	0.98801 (13)	0.0273 (10)	
H40	0.6276	0.2771	0.9757	0.033*	
C41	0.7653 (4)	0.2262 (3)	1.00714 (14)	0.0301 (10)	
H41	0.7345	0.1687	1.0079	0.036*	
C42	0.8727 (4)	0.2465 (3)	1.02483 (14)	0.0321 (11)	
H42	0.9151	0.2034	1.0384	0.039*	
C43	0.9184 (4)	0.3294 (3)	1.02275 (13)	0.0330 (11)	
H43	0.9939	0.3420	1.0344	0.040*	
C44	0.8582 (4)	0.3948 (3)	1.00422 (13)	0.0290 (10)	
H44	0.8912	0.4516	1.0032	0.035*	
C1S	0.6579 (7)	0.5532 (4)	0.85218 (17)	0.0546 (16)	
H1S	0.7135	0.5172	0.8392	0.066*	
C2S	0.5853 (9)	0.6966 (4)	0.8653 (2)	0.075 (2)	
H2S1	0.5209	0.7172	0.8487	0.112*	
H2S2	0.6338	0.7460	0.8738	0.112*	
H2S3	0.5484	0.6674	0.8866	0.112*	
C3S	0.7590 (17)	0.6723 (8)	0.8206 (3)	0.162 (7)	
H3S1	0.8205	0.6275	0.8161	0.244*	
H3S2	0.7977	0.7227	0.8321	0.244*	
H3S3	0.7219	0.6895	0.7971	0.244*	
C4S	0.5363 (6)	0.1985 (6)	0.89094 (18)	0.0617 (19)	
H4S	0.4583	0.2126	0.8813	0.074*	
C5S	0.4706 (8)	0.0542 (6)	0.8859 (3)	0.087 (3)	
H5S1	0.4443	0.0230	0.9081	0.130*	
H5S2	0.5028	0.0130	0.8679	0.130*	
H5S3	0.4009	0.0850	0.8753	0.130*	
C6S	0.6682 (7)	0.0843 (5)	0.9135 (2)	0.076 (2)	
H6S1	0.7145	0.1340	0.9227	0.114*	
H6S2	0.7185	0.0516	0.8960	0.114*	
H6S3	0.6454	0.0468	0.9342	0.114*	
C7S	0.7337 (14)	0.0738 (12)	0.7958 (4)	0.073 (4)	0.50
H7S	0.7310	0.0400	0.7738	0.087*	0.50
C8S	0.8177 (13)	0.2045 (12)	0.8203 (6)	0.088 (6)	0.50
H8S1	0.8742	0.1824	0.8390	0.132*	0.50
H8S2	0.7387	0.2167	0.8318	0.132*	0.50
H8S3	0.8506	0.2578	0.8095	0.132*	0.50
C9S	0.8683 (14)	0.1656 (15)	0.7617 (5)	0.090 (6)	0.50
H9S1	0.8114	0.1825	0.7421	0.136*	0.50
H9S2	0.9158	0.1153	0.7536	0.136*	0.50
H9S3	0.9234	0.2139	0.7670	0.136*	0.50
C10S	−0.0132 (11)	0.3143 (10)	0.6572 (3)	0.072 (4)	0.50
H10S	0.0234	0.2620	0.6654	0.087*	0.50
C11S	0.0111 (14)	0.4612 (9)	0.6631 (3)	0.073 (5)	0.50
H11C	−0.0497	0.4605	0.6433	0.110*	0.50
H11D	0.0820	0.4952	0.6553	0.110*	0.50
H11E	−0.0245	0.4874	0.6853	0.110*	0.50
C12S	0.1468 (17)	0.3777 (13)	0.6934 (5)	0.211 (17)	0.50
H12C	0.1209	0.3913	0.7187	0.317*	0.50
H12D	0.2027	0.4227	0.6846	0.317*	0.50
H12E	0.1883	0.3215	0.6931	0.317*	0.50
C13S	0.1600 (6)	0.5598 (4)	0.96013 (19)	0.0507 (15)	
H13S	0.0938	0.5719	0.9763	0.061*	
C14S	0.2130 (8)	0.7101 (4)	0.9664 (2)	0.0675 (19)	
H14A	0.1344	0.7110	0.9793	0.101*	
H14B	0.2118	0.7522	0.9462	0.101*	
H14C	0.2781	0.7251	0.9838	0.101*	
C15S	0.3432 (7)	0.6118 (5)	0.92843 (18)	0.0641 (18)	
H15A	0.3502	0.5508	0.9214	0.096*	
H15B	0.4160	0.6292	0.9423	0.096*	
H15C	0.3359	0.6476	0.9062	0.096*	
Cl1	0.50260 (15)	0.85461 (10)	0.94046 (5)	0.0593 (4)	
Cl2	0.9939 (3)	0.4579 (3)	0.78860 (11)	0.0913 (10)	0.75
Fe1	0.58549 (6)	0.38649 (5)	0.885291 (19)	0.02820 (18)	
N1	0.1873 (4)	0.3786 (3)	0.86586 (11)	0.0339 (9)	
H1N	0.1305	0.4054	0.8531	0.041*	
N2	0.9789 (3)	0.3981 (3)	0.91209 (11)	0.0281 (8)	
H2N	1.0350	0.4311	0.9223	0.034*	
Cl2B	0.9774 (11)	0.5106 (10)	0.7963 (4)	0.110 (3)	0.25
N1S	0.6622 (7)	0.6372 (4)	0.84593 (15)	0.0696 (18)	
N2S	0.5616 (7)	0.1139 (4)	0.89539 (18)	0.0749 (19)	
N3S	0.8031 (10)	0.1444 (9)	0.7934 (3)	0.062 (3)	0.50
N4S	0.0485 (10)	0.3745 (7)	0.6712 (2)	0.058 (3)	0.50
N5S	0.2350 (5)	0.6237 (3)	0.95160 (14)	0.0476 (12)	
O1	0.5973 (3)	0.3611 (3)	0.82730 (10)	0.0453 (9)	
O2	0.3882 (3)	0.3688 (3)	0.87832 (10)	0.0365 (8)	
O3	0.5607 (3)	0.4197 (2)	0.94070 (9)	0.0315 (8)	
O4	0.7818 (3)	0.3839 (2)	0.89566 (9)	0.0336 (8)	
O1A	0.4093 (5)	0.8463 (4)	0.91396 (16)	0.0812 (16)	
O2A	0.5138 (5)	0.7759 (3)	0.96162 (16)	0.0766 (15)	
O3A	0.4698 (7)	0.9222 (4)	0.96452 (19)	0.105 (2)	
O4A	0.6165 (5)	0.8726 (3)	0.9218 (2)	0.095 (2)	
O5A	0.9724 (9)	0.3674 (6)	0.7799 (4)	0.139 (4)	0.75
O6A	0.9855 (6)	0.4607 (5)	0.82655 (17)	0.112 (2)	
O7A	0.9060 (7)	0.5084 (5)	0.7701 (2)	0.117 (2)	
O8A	1.1074 (7)	0.4787 (6)	0.7775 (3)	0.130 (2)	
O9A	0.990 (3)	0.5906 (16)	0.8090 (8)	0.111 (9)	0.25
O1S	0.5875 (3)	0.5186 (2)	0.87352 (9)	0.0377 (8)	
O2S	0.6001 (4)	0.2549 (3)	0.89770 (12)	0.0531 (11)	
O3S	0.6748 (11)	0.0458 (9)	0.8204 (3)	0.085 (3)	0.50
O4S	−0.0973 (11)	0.2932 (8)	0.6382 (3)	0.092 (4)	0.50
O5S	0.1713 (4)	0.4836 (3)	0.94803 (15)	0.0652 (13)	
P1	0.50025 (13)	0.37469 (10)	0.79961 (4)	0.0402 (3)	
P2	0.65128 (10)	0.45737 (8)	0.96670 (3)	0.0258 (3)	

### Atomic displacement parameters (Å^2^)

**Table:**

	*U*^11^	*U*^22^	*U*^33^	*U*^12^	*U*^13^	*U*^23^
C1	0.034 (3)	0.032 (3)	0.039 (3)	−0.005 (2)	0.009 (2)	−0.008 (2)
C2	0.037 (3)	0.031 (3)	0.051 (3)	−0.002 (2)	0.004 (2)	0.000 (3)
C3	0.058 (4)	0.036 (4)	0.073 (4)	−0.001 (3)	0.023 (3)	0.007 (3)
C4	0.078 (5)	0.032 (4)	0.102 (6)	0.009 (3)	0.019 (4)	0.012 (4)
C5	0.078 (5)	0.043 (4)	0.080 (5)	−0.005 (4)	0.021 (4)	0.021 (4)
C6	0.058 (4)	0.043 (4)	0.059 (4)	−0.010 (3)	0.022 (3)	0.003 (3)
C7	0.047 (3)	0.036 (3)	0.037 (3)	−0.005 (3)	0.003 (2)	0.001 (2)
C8	0.025 (2)	0.034 (3)	0.035 (3)	0.002 (2)	−0.001 (2)	0.003 (2)
C9	0.028 (2)	0.027 (3)	0.033 (2)	0.002 (2)	0.0004 (19)	−0.001 (2)
C10	0.030 (2)	0.035 (3)	0.043 (3)	−0.004 (2)	0.007 (2)	0.002 (2)
C11	0.042 (3)	0.029 (3)	0.044 (3)	0.000 (2)	−0.003 (2)	0.001 (2)
C12	0.040 (3)	0.027 (3)	0.048 (3)	0.005 (2)	0.000 (2)	−0.005 (2)
C13	0.036 (3)	0.032 (3)	0.044 (3)	0.009 (2)	0.005 (2)	0.003 (2)
C14	0.023 (2)	0.028 (3)	0.030 (2)	−0.0010 (19)	0.0018 (19)	0.005 (2)
C15	0.024 (2)	0.026 (3)	0.032 (2)	−0.0004 (19)	0.0040 (19)	0.005 (2)
C16	0.033 (2)	0.031 (3)	0.051 (3)	0.004 (2)	0.011 (2)	0.005 (2)
C17	0.042 (3)	0.025 (3)	0.060 (3)	−0.004 (2)	0.008 (3)	0.006 (3)
C18	0.045 (3)	0.017 (3)	0.064 (4)	0.003 (2)	0.008 (3)	0.002 (3)
C19	0.032 (2)	0.025 (3)	0.047 (3)	0.000 (2)	0.003 (2)	0.007 (2)
C20	0.026 (2)	0.018 (2)	0.038 (2)	−0.0020 (19)	0.0053 (19)	0.003 (2)
C21	0.041 (3)	0.036 (3)	0.034 (3)	−0.003 (2)	0.003 (2)	−0.003 (2)
C22	0.062 (4)	0.050 (4)	0.044 (3)	−0.015 (3)	0.003 (3)	−0.012 (3)
C23	0.068 (4)	0.076 (6)	0.055 (4)	−0.024 (4)	−0.003 (3)	−0.016 (4)
C24	0.084 (5)	0.058 (5)	0.048 (4)	−0.034 (4)	0.015 (3)	−0.013 (3)
C25	0.083 (5)	0.041 (4)	0.048 (3)	−0.011 (3)	0.011 (3)	−0.005 (3)
C26	0.050 (3)	0.046 (4)	0.049 (3)	−0.002 (3)	0.010 (3)	−0.001 (3)
C27	0.071 (4)	0.033 (3)	0.050 (3)	0.003 (3)	0.015 (3)	−0.005 (3)
C28	0.095 (5)	0.049 (4)	0.045 (3)	−0.013 (4)	0.007 (3)	0.006 (3)
C29	0.136 (8)	0.042 (4)	0.056 (4)	−0.023 (5)	0.014 (4)	−0.010 (4)
C30	0.121 (7)	0.063 (5)	0.061 (4)	0.013 (6)	0.023 (5)	−0.006 (4)
C31	0.093 (6)	0.139 (10)	0.068 (5)	0.017 (7)	0.038 (5)	0.022 (6)
C32	0.069 (5)	0.105 (7)	0.054 (4)	−0.004 (5)	0.025 (3)	0.002 (5)
C33	0.032 (2)	0.019 (2)	0.035 (2)	0.0056 (19)	0.010 (2)	0.008 (2)
C34	0.036 (3)	0.029 (3)	0.043 (3)	0.006 (2)	0.006 (2)	0.010 (2)
C35	0.039 (3)	0.037 (3)	0.060 (4)	0.018 (2)	0.014 (3)	0.015 (3)
C36	0.069 (4)	0.028 (3)	0.044 (3)	0.014 (3)	0.015 (3)	0.010 (3)
C37	0.067 (4)	0.020 (3)	0.047 (3)	0.006 (3)	0.000 (3)	−0.003 (2)
C38	0.040 (3)	0.023 (2)	0.047 (3)	0.001 (2)	0.003 (2)	−0.004 (2)
C39	0.023 (2)	0.021 (2)	0.031 (2)	0.0029 (18)	0.0029 (17)	0.003 (2)
C40	0.024 (2)	0.022 (2)	0.036 (3)	0.0026 (18)	−0.0012 (19)	0.003 (2)
C41	0.033 (2)	0.018 (2)	0.039 (3)	0.0000 (19)	0.004 (2)	0.004 (2)
C42	0.026 (2)	0.031 (3)	0.039 (3)	0.007 (2)	0.0063 (19)	0.010 (2)
C43	0.024 (2)	0.033 (3)	0.042 (3)	0.003 (2)	−0.011 (2)	−0.004 (2)
C44	0.029 (2)	0.022 (2)	0.036 (2)	0.000 (2)	−0.0024 (19)	−0.003 (2)
C1S	0.075 (4)	0.046 (4)	0.043 (3)	−0.012 (3)	0.001 (3)	0.001 (3)
C2S	0.120 (6)	0.030 (4)	0.074 (5)	−0.011 (4)	−0.018 (5)	0.008 (4)
C3S	0.293 (19)	0.112 (10)	0.082 (7)	−0.093 (12)	0.047 (10)	0.022 (7)
C4S	0.045 (3)	0.090 (6)	0.050 (4)	0.008 (4)	−0.009 (3)	0.006 (4)
C5S	0.086 (5)	0.081 (6)	0.094 (6)	0.028 (5)	−0.019 (5)	0.001 (5)
C6S	0.074 (5)	0.066 (5)	0.089 (5)	−0.029 (4)	0.024 (4)	−0.027 (5)
C7S	0.058 (8)	0.099 (14)	0.061 (9)	−0.007 (9)	0.004 (7)	−0.003 (9)
C8S	0.047 (8)	0.094 (13)	0.124 (14)	0.032 (8)	−0.027 (9)	−0.042 (12)
C9S	0.058 (8)	0.138 (18)	0.075 (10)	−0.026 (10)	0.008 (8)	−0.036 (11)
C10S	0.099 (9)	0.083 (9)	0.036 (6)	0.057 (8)	−0.012 (6)	−0.012 (6)
C11S	0.076 (9)	0.101 (12)	0.044 (7)	−0.007 (9)	0.021 (7)	0.049 (8)
C12S	0.25 (3)	0.079 (15)	0.31 (3)	0.088 (18)	−0.19 (3)	−0.12 (2)
C13S	0.043 (3)	0.038 (4)	0.071 (4)	0.001 (3)	−0.021 (3)	−0.006 (3)
C14S	0.088 (5)	0.036 (4)	0.079 (5)	0.002 (3)	0.009 (4)	−0.012 (4)
C15S	0.087 (5)	0.051 (4)	0.054 (4)	0.005 (4)	0.002 (4)	0.005 (4)
Cl1	0.0557 (9)	0.0319 (8)	0.0903 (12)	0.0051 (7)	−0.0153 (9)	−0.0077 (8)
Cl2	0.0628 (16)	0.104 (3)	0.107 (2)	−0.0066 (18)	−0.0185 (16)	0.028 (2)
Fe1	0.0276 (3)	0.0214 (3)	0.0356 (3)	−0.0015 (3)	0.0017 (3)	0.0005 (3)
N1	0.032 (2)	0.031 (2)	0.039 (2)	0.0011 (18)	0.0006 (17)	0.005 (2)
N2	0.0216 (18)	0.026 (2)	0.037 (2)	−0.0006 (16)	0.0002 (15)	−0.0010 (18)
Cl2B	0.103 (5)	0.115 (5)	0.111 (5)	0.028 (5)	−0.023 (4)	0.018 (5)
N1S	0.124 (5)	0.038 (3)	0.046 (3)	−0.025 (3)	0.014 (3)	0.007 (3)
N2S	0.118 (5)	0.031 (3)	0.075 (4)	0.028 (4)	−0.008 (4)	0.007 (3)
N3S	0.048 (6)	0.088 (9)	0.050 (6)	0.000 (6)	−0.004 (5)	−0.009 (6)
N4S	0.107 (8)	0.054 (7)	0.013 (4)	0.052 (6)	−0.001 (4)	−0.001 (4)
N5S	0.056 (3)	0.031 (3)	0.055 (3)	0.002 (2)	−0.009 (2)	−0.006 (2)
O1	0.046 (2)	0.045 (2)	0.045 (2)	−0.0051 (18)	0.0048 (18)	−0.0127 (18)
O2	0.0233 (15)	0.043 (2)	0.0430 (19)	−0.0015 (14)	−0.0017 (14)	−0.0011 (17)
O3	0.0261 (16)	0.0281 (18)	0.0402 (18)	0.0027 (13)	0.0025 (14)	0.0031 (16)
O4	0.0277 (15)	0.0276 (18)	0.0455 (19)	0.0006 (15)	0.0011 (14)	0.0009 (17)
O1A	0.078 (3)	0.066 (3)	0.099 (4)	−0.015 (3)	−0.027 (3)	0.024 (3)
O2A	0.091 (4)	0.039 (3)	0.100 (4)	0.016 (3)	−0.018 (3)	0.006 (3)
O3A	0.156 (6)	0.054 (3)	0.105 (4)	0.052 (4)	−0.033 (4)	−0.029 (3)
O4A	0.059 (3)	0.041 (3)	0.186 (7)	0.000 (2)	0.014 (4)	0.002 (4)
O5A	0.112 (7)	0.071 (6)	0.232 (13)	−0.002 (5)	−0.055 (8)	−0.027 (7)
O6A	0.103 (4)	0.155 (5)	0.077 (3)	0.025 (4)	−0.014 (3)	0.050 (4)
O7A	0.103 (4)	0.129 (5)	0.119 (4)	0.024 (4)	−0.043 (4)	0.025 (4)
O8A	0.093 (4)	0.133 (5)	0.164 (5)	0.009 (4)	−0.001 (4)	−0.017 (5)
O9A	0.14 (2)	0.065 (16)	0.13 (2)	0.018 (15)	0.002 (18)	−0.053 (15)
O1S	0.0430 (19)	0.032 (2)	0.0384 (18)	−0.0045 (17)	−0.0060 (17)	0.0061 (16)
O2S	0.062 (3)	0.031 (2)	0.066 (3)	−0.018 (2)	0.015 (2)	−0.004 (2)
O3S	0.089 (8)	0.100 (9)	0.068 (6)	−0.012 (7)	0.003 (6)	−0.004 (7)
O4S	0.099 (8)	0.079 (8)	0.098 (8)	0.008 (7)	0.030 (7)	0.010 (7)
O5S	0.050 (2)	0.040 (3)	0.106 (4)	0.000 (2)	−0.026 (2)	−0.024 (3)
P1	0.0423 (7)	0.0364 (8)	0.0421 (7)	−0.0046 (6)	0.0086 (6)	−0.0012 (7)
P2	0.0226 (5)	0.0194 (6)	0.0353 (6)	0.0015 (5)	0.0030 (5)	0.0031 (5)

### Geometric parameters (Å, °)

**Table:**

C1—O2	1.241 (6)	C41—C42	1.378 (7)
C1—N1	1.325 (6)	C41—H41	0.9500
C1—C2	1.506 (8)	C42—C43	1.377 (7)
C2—C3	1.361 (8)	C42—H42	0.9500
C2—C7	1.388 (8)	C43—C44	1.381 (7)
C3—C4	1.397 (9)	C43—H43	0.9500
C3—H3	0.9500	C44—H44	0.9500
C4—C5	1.390 (10)	C1S—O1S	1.216 (7)
C4—H4	0.9500	C1S—N1S	1.318 (9)
C5—C6	1.355 (10)	C1S—H1S	0.9500
C5—H5	0.9500	C2S—N1S	1.431 (10)
C6—C7	1.421 (8)	C2S—H2S1	0.9800
C6—H6	0.9500	C2S—H2S2	0.9800
C7—P1	1.808 (6)	C2S—H2S3	0.9800
C8—N1	1.474 (6)	C3S—N1S	1.503 (13)
C8—C13	1.519 (7)	C3S—H3S1	0.9800
C8—C9	1.536 (6)	C3S—H3S2	0.9800
C8—H8	1.0000	C3S—H3S3	0.9800
C9—N2^i^	1.454 (6)	C4S—O2S	1.144 (8)
C9—C10	1.518 (7)	C4S—N2S	1.344 (10)
C9—H9	1.0000	C4S—H4S	0.9500
C10—C11	1.510 (8)	C5S—N2S	1.401 (11)
C10—H10A	0.9900	C5S—H5S1	0.9800
C10—H10B	0.9900	C5S—H5S2	0.9800
C11—C12	1.513 (8)	C5S—H5S3	0.9800
C11—H11A	0.9900	C6S—N2S	1.416 (11)
C11—H11B	0.9900	C6S—H6S1	0.9800
C12—C13	1.518 (8)	C6S—H6S2	0.9800
C12—H12A	0.9900	C6S—H6S3	0.9800
C12—H12B	0.9900	C7S—O3S	1.185 (17)
C13—H13A	0.9900	C7S—N3S	1.33 (2)
C13—H13B	0.9900	C7S—H7S	0.9500
C14—O4	1.247 (6)	C8S—N3S	1.35 (2)
C14—N2	1.323 (6)	C8S—H8S1	0.9800
C14—C15	1.485 (7)	C8S—H8S2	0.9800
C15—C16	1.394 (7)	C8S—H8S3	0.9800
C15—C20	1.419 (6)	C9S—N3S	1.39 (2)
C16—C17	1.376 (8)	C9S—H9S1	0.9800
C16—H16	0.9500	C9S—H9S2	0.9800
C17—C18	1.389 (8)	C9S—H9S3	0.9800
C17—H17	0.9500	C10S—O4S	1.196 (14)
C18—C19	1.388 (7)	C10S—N4S	1.256 (14)
C18—H18	0.9500	C10S—H10S	0.9500
C19—C20	1.403 (7)	C11S—N4S	1.431 (14)
C19—H19	0.9500	C11S—H11C	0.9800
C20—P2	1.804 (5)	C11S—H11D	0.9800
C21—C22	1.377 (9)	C11S—H11E	0.9800
C21—C26	1.386 (8)	C12S—N4S	1.347 (16)
C21—P1	1.808 (6)	C12S—H12C	0.9800
C22—C23	1.375 (10)	C12S—H12D	0.9800
C22—H22	0.9500	C12S—H12E	0.9800
C23—C24	1.367 (11)	C13S—O5S	1.262 (7)
C23—H23	0.9500	C13S—N5S	1.322 (8)
C24—C25	1.354 (11)	C13S—H13S	0.9500
C24—H24	0.9500	C14S—N5S	1.457 (8)
C25—C26	1.378 (9)	C14S—H14A	0.9800
C25—H25	0.9500	C14S—H14B	0.9800
C26—H26	0.9500	C14S—H14C	0.9800
C27—C32	1.365 (10)	C15S—N5S	1.466 (9)
C27—C28	1.423 (10)	C15S—H15A	0.9800
C27—P1	1.800 (6)	C15S—H15B	0.9800
C28—C29	1.368 (10)	C15S—H15C	0.9800
C28—H28	0.9500	Cl1—O3A	1.406 (6)
C29—C30	1.321 (13)	Cl1—O1A	1.410 (5)
C29—H29	0.9500	Cl1—O2A	1.442 (5)
C30—C31	1.424 (13)	Cl1—O4A	1.447 (6)
C30—H30	0.9500	Cl2—Cl2B	0.878 (14)
C31—C32	1.429 (10)	Cl2—O8A	1.347 (8)
C31—H31	0.9500	Cl2—O6A	1.378 (7)
C32—H32	0.9500	Cl2—O7A	1.409 (7)
C33—C38	1.375 (7)	Cl2—O5A	1.453 (9)
C33—C34	1.402 (7)	Fe1—O1S	2.084 (4)
C33—P2	1.816 (5)	Fe1—O2S	2.087 (4)
C34—C35	1.430 (8)	Fe1—O3	2.089 (3)
C34—H34	0.9500	Fe1—O1	2.140 (4)
C35—C36	1.354 (9)	Fe1—O4	2.187 (3)
C35—H35	0.9500	Fe1—O2	2.197 (3)
C36—C37	1.378 (9)	N1—H1N	0.8800
C36—H36	0.9500	N2—C9^ii^	1.454 (6)
C37—C38	1.372 (8)	N2—H2N	0.8800
C37—H37	0.9500	Cl2B—O7A	1.229 (14)
C38—H38	0.9500	Cl2B—O9A	1.32 (3)
C39—C40	1.392 (7)	Cl2B—O6A	1.344 (14)
C39—C44	1.408 (6)	Cl2B—O8A	1.656 (13)
C39—P2	1.801 (5)	O1—P1	1.478 (4)
C40—C41	1.401 (6)	O3—P2	1.488 (4)
C40—H40	0.9500		
			
O2—C1—N1	122.9 (5)	N1S—C2S—H2S2	109.5
O2—C1—C2	120.9 (4)	H2S1—C2S—H2S2	109.5
N1—C1—C2	116.1 (5)	N1S—C2S—H2S3	109.5
C3—C2—C7	121.3 (5)	H2S1—C2S—H2S3	109.5
C3—C2—C1	118.5 (5)	H2S2—C2S—H2S3	109.5
C7—C2—C1	120.1 (5)	N1S—C3S—H3S1	109.5
C2—C3—C4	120.5 (6)	N1S—C3S—H3S2	109.5
C2—C3—H3	119.8	H3S1—C3S—H3S2	109.5
C4—C3—H3	119.8	N1S—C3S—H3S3	109.5
C5—C4—C3	119.2 (7)	H3S1—C3S—H3S3	109.5
C5—C4—H4	120.4	H3S2—C3S—H3S3	109.5
C3—C4—H4	120.4	O2S—C4S—N2S	126.1 (7)
C6—C5—C4	120.2 (6)	O2S—C4S—H4S	117.0
C6—C5—H5	119.9	N2S—C4S—H4S	117.0
C4—C5—H5	119.9	N2S—C5S—H5S1	109.5
C5—C6—C7	121.3 (6)	N2S—C5S—H5S2	109.5
C5—C6—H6	119.4	H5S1—C5S—H5S2	109.5
C7—C6—H6	119.4	N2S—C5S—H5S3	109.5
C2—C7—C6	117.5 (5)	H5S1—C5S—H5S3	109.5
C2—C7—P1	123.7 (4)	H5S2—C5S—H5S3	109.5
C6—C7—P1	118.6 (4)	N2S—C6S—H6S1	109.5
N1—C8—C13	112.0 (4)	N2S—C6S—H6S2	109.5
N1—C8—C9	110.8 (4)	H6S1—C6S—H6S2	109.5
C13—C8—C9	109.1 (4)	N2S—C6S—H6S3	109.5
N1—C8—H8	108.3	H6S1—C6S—H6S3	109.5
C13—C8—H8	108.3	H6S2—C6S—H6S3	109.5
C9—C8—H8	108.3	O3S—C7S—N3S	131.2 (16)
N2^i^—C9—C10	111.9 (4)	O3S—C7S—H7S	114.4
N2^i^—C9—C8	110.2 (4)	N3S—C7S—H7S	114.4
C10—C9—C8	109.4 (4)	N3S—C8S—H8S1	109.5
N2^i^—C9—H9	108.4	N3S—C8S—H8S2	109.5
C10—C9—H9	108.4	H8S1—C8S—H8S2	109.5
C8—C9—H9	108.4	N3S—C8S—H8S3	109.5
C11—C10—C9	112.0 (4)	H8S1—C8S—H8S3	109.5
C11—C10—H10A	109.2	H8S2—C8S—H8S3	109.5
C9—C10—H10A	109.2	N3S—C9S—H9S1	109.5
C11—C10—H10B	109.2	N3S—C9S—H9S2	109.5
C9—C10—H10B	109.2	H9S1—C9S—H9S2	109.5
H10A—C10—H10B	107.9	N3S—C9S—H9S3	109.5
C10—C11—C12	111.9 (4)	H9S1—C9S—H9S3	109.5
C10—C11—H11A	109.2	H9S2—C9S—H9S3	109.5
C12—C11—H11A	109.2	O4S—C10S—N4S	148.1 (15)
C10—C11—H11B	109.2	O4S—C10S—H10S	106.0
C12—C11—H11B	109.2	N4S—C10S—H10S	106.0
H11A—C11—H11B	107.9	N4S—C11S—H11C	109.5
C11—C12—C13	110.8 (4)	N4S—C11S—H11D	109.5
C11—C12—H12A	109.5	H11C—C11S—H11D	109.5
C13—C12—H12A	109.5	N4S—C11S—H11E	109.5
C11—C12—H12B	109.5	H11C—C11S—H11E	109.5
C13—C12—H12B	109.5	H11D—C11S—H11E	109.5
H12A—C12—H12B	108.1	N4S—C12S—H12C	109.5
C12—C13—C8	111.8 (4)	N4S—C12S—H12D	109.5
C12—C13—H13A	109.3	H12C—C12S—H12D	109.5
C8—C13—H13A	109.3	N4S—C12S—H12E	109.5
C12—C13—H13B	109.3	H12C—C12S—H12E	109.5
C8—C13—H13B	109.3	H12D—C12S—H12E	109.5
H13A—C13—H13B	107.9	O5S—C13S—N5S	123.6 (6)
O4—C14—N2	122.5 (5)	O5S—C13S—H13S	118.2
O4—C14—C15	121.2 (4)	N5S—C13S—H13S	118.2
N2—C14—C15	116.3 (4)	N5S—C14S—H14A	109.5
C16—C15—C20	119.1 (5)	N5S—C14S—H14B	109.5
C16—C15—C14	118.5 (4)	H14A—C14S—H14B	109.5
C20—C15—C14	122.2 (4)	N5S—C14S—H14C	109.5
C17—C16—C15	121.5 (5)	H14A—C14S—H14C	109.5
C17—C16—H16	119.2	H14B—C14S—H14C	109.5
C15—C16—H16	119.2	N5S—C15S—H15A	109.5
C16—C17—C18	119.7 (5)	N5S—C15S—H15B	109.5
C16—C17—H17	120.1	H15A—C15S—H15B	109.5
C18—C17—H17	120.1	N5S—C15S—H15C	109.5
C19—C18—C17	120.2 (5)	H15A—C15S—H15C	109.5
C19—C18—H18	119.9	H15B—C15S—H15C	109.5
C17—C18—H18	119.9	O3A—Cl1—O1A	107.6 (4)
C18—C19—C20	120.8 (5)	O3A—Cl1—O2A	108.6 (4)
C18—C19—H19	119.6	O1A—Cl1—O2A	110.3 (3)
C20—C19—H19	119.6	O3A—Cl1—O4A	111.5 (4)
C19—C20—C15	118.6 (4)	O1A—Cl1—O4A	109.1 (4)
C19—C20—P2	119.4 (3)	O2A—Cl1—O4A	109.6 (4)
C15—C20—P2	121.9 (4)	Cl2B—Cl2—O8A	93.7 (10)
C22—C21—C26	119.4 (6)	Cl2B—Cl2—O6A	69.1 (10)
C22—C21—P1	123.1 (5)	O8A—Cl2—O6A	110.7 (5)
C26—C21—P1	117.1 (4)	Cl2B—Cl2—O7A	59.8 (10)
C23—C22—C21	120.0 (7)	O8A—Cl2—O7A	111.1 (6)
C23—C22—H22	120.0	O6A—Cl2—O7A	114.3 (5)
C21—C22—H22	120.0	Cl2B—Cl2—O5A	157.9 (12)
C24—C23—C22	120.7 (7)	O8A—Cl2—O5A	108.3 (7)
C24—C23—H23	119.6	O6A—Cl2—O5A	103.5 (7)
C22—C23—H23	119.6	O7A—Cl2—O5A	108.6 (6)
C25—C24—C23	119.2 (7)	O1S—Fe1—O2S	174.95 (17)
C25—C24—H24	120.4	O1S—Fe1—O3	87.58 (14)
C23—C24—H24	120.4	O2S—Fe1—O3	92.41 (15)
C24—C25—C26	121.6 (7)	O1S—Fe1—O1	88.75 (15)
C24—C25—H25	119.2	O2S—Fe1—O1	91.61 (17)
C26—C25—H25	119.2	O3—Fe1—O1	174.52 (15)
C25—C26—C21	119.0 (6)	O1S—Fe1—O4	92.43 (14)
C25—C26—H26	120.5	O2S—Fe1—O4	82.52 (16)
C21—C26—H26	120.5	O3—Fe1—O4	88.16 (13)
C32—C27—C28	119.9 (6)	O1—Fe1—O4	96.06 (14)
C32—C27—P1	118.8 (5)	O1S—Fe1—O2	96.26 (14)
C28—C27—P1	121.1 (5)	O2S—Fe1—O2	88.79 (16)
C29—C28—C27	119.5 (8)	O3—Fe1—O2	90.72 (13)
C29—C28—H28	120.3	O1—Fe1—O2	85.63 (14)
C27—C28—H28	120.3	O4—Fe1—O2	171.18 (14)
C30—C29—C28	122.3 (9)	C1—N1—C8	123.0 (4)
C30—C29—H29	118.9	C1—N1—H1N	118.5
C28—C29—H29	118.9	C8—N1—H1N	118.5
C29—C30—C31	120.5 (8)	C14—N2—C9^ii^	121.8 (4)
C29—C30—H30	119.7	C14—N2—H2N	119.1
C31—C30—H30	119.7	C9^ii^—N2—H2N	119.1
C30—C31—C32	118.4 (8)	Cl2—Cl2B—O7A	82.1 (13)
C30—C31—H31	120.8	Cl2—Cl2B—O9A	162 (2)
C32—C31—H31	120.8	O7A—Cl2B—O9A	110.9 (17)
C27—C32—C31	119.4 (8)	Cl2—Cl2B—O6A	73.3 (11)
C27—C32—H32	120.3	O7A—Cl2B—O6A	130.9 (14)
C31—C32—H32	120.3	O9A—Cl2B—O6A	104.2 (17)
C38—C33—C34	122.1 (5)	Cl2—Cl2B—O8A	54.3 (8)
C38—C33—P2	121.4 (4)	O7A—Cl2B—O8A	103.0 (10)
C34—C33—P2	116.5 (4)	O9A—Cl2B—O8A	109.5 (17)
C33—C34—C35	116.5 (5)	O6A—Cl2B—O8A	96.2 (8)
C33—C34—H34	121.8	C1S—N1S—C2S	121.8 (6)
C35—C34—H34	121.8	C1S—N1S—C3S	119.0 (8)
C36—C35—C34	120.6 (5)	C2S—N1S—C3S	119.0 (8)
C36—C35—H35	119.7	C4S—N2S—C5S	117.5 (7)
C34—C35—H35	119.7	C4S—N2S—C6S	122.6 (7)
C35—C36—C37	120.9 (6)	C5S—N2S—C6S	119.3 (6)
C35—C36—H36	119.6	C7S—N3S—C8S	125.5 (14)
C37—C36—H36	119.6	C7S—N3S—C9S	122.7 (14)
C38—C37—C36	120.6 (6)	C8S—N3S—C9S	111.8 (15)
C38—C37—H37	119.7	C10S—N4S—C12S	134.4 (13)
C36—C37—H37	119.7	C10S—N4S—C11S	117.1 (11)
C37—C38—C33	119.3 (5)	C12S—N4S—C11S	108.5 (13)
C37—C38—H38	120.4	C13S—N5S—C14S	119.6 (6)
C33—C38—H38	120.4	C13S—N5S—C15S	123.0 (6)
C40—C39—C44	119.8 (4)	C14S—N5S—C15S	117.4 (6)
C40—C39—P2	117.7 (3)	P1—O1—Fe1	126.6 (2)
C44—C39—P2	122.2 (4)	C1—O2—Fe1	138.8 (3)
C39—C40—C41	120.2 (4)	P2—O3—Fe1	128.09 (19)
C39—C40—H40	119.9	C14—O4—Fe1	142.0 (3)
C41—C40—H40	119.9	Cl2B—O6A—Cl2	37.6 (6)
C42—C41—C40	119.6 (5)	Cl2B—O7A—Cl2	38.1 (7)
C42—C41—H41	120.2	Cl2—O8A—Cl2B	32.0 (6)
C40—C41—H41	120.2	C1S—O1S—Fe1	124.5 (4)
C43—C42—C41	119.8 (5)	C4S—O2S—Fe1	130.5 (5)
C43—C42—H42	120.1	O1—P1—C27	111.1 (3)
C41—C42—H42	120.1	O1—P1—C21	113.3 (3)
C42—C43—C44	122.2 (4)	C27—P1—C21	102.8 (3)
C42—C43—H43	118.9	O1—P1—C7	113.7 (2)
C44—C43—H43	118.9	C27—P1—C7	107.0 (3)
C43—C44—C39	118.3 (5)	C21—P1—C7	108.3 (3)
C43—C44—H44	120.8	O3—P2—C39	111.7 (2)
C39—C44—H44	120.8	O3—P2—C20	114.8 (2)
O1S—C1S—N1S	124.3 (7)	C39—P2—C20	108.0 (2)
O1S—C1S—H1S	117.8	O3—P2—C33	109.2 (2)
N1S—C1S—H1S	117.8	C39—P2—C33	105.5 (2)
N1S—C2S—H2S1	109.5	C20—P2—C33	107.1 (2)

Symmetry codes: (i) *x*−1, *y*, *z*; (ii) *x*+1, *y*, *z*.
